# Graphene Quantum Dot-TiO_2_ Photonic Crystal Films for Photocatalytic Applications

**DOI:** 10.3390/nano10122566

**Published:** 2020-12-21

**Authors:** Maria-Athina Apostolaki, Alexia Toumazatou, Maria Antoniadou, Elias Sakellis, Evangelia Xenogiannopoulou, Spiros Gardelis, Nikos Boukos, Polycarpos Falaras, Athanasios Dimoulas, Vlassis Likodimos

**Affiliations:** 1 Section of Condensed Matter Physics, Department of Physics, National and Kapodistrian University of Athens, Panepistimiopolis Zografou, GR-15784 Athens, Greece; marapos@phys.uoa.gr (M.-A.A.); alextoum@phys.uoa.gr (A.T.); sgardelis@phys.uoa.gr (S.G.); 2 Institute of Nanoscience and Nanotechnology, National Center for Scientific Research “Demokritos”, Agia Paraskevi, 15341 Athens, Greece; m.antoniadou@inn.demokritos.gr (M.A.); e.sakellis@inn.demokritos.gr (E.S.); e.xenogiannopoulou@inn.demokritos.gr (E.X.); n.boukos@inn.demokritos.gr (N.B.); p.falaras@inn.demokritos.gr (P.F.); a.dimoulas@inn.demokritos.gr (A.D.)

**Keywords:** graphene quantum dots, photonic crystals, titanium dioxide, photocatalysis, slow photons

## Abstract

Photonic crystal structuring has emerged as an advanced method to enhance solar light harvesting by metal oxide photocatalysts along with rational compositional modifications of the materials’ properties. In this work, surface functionalization of TiO_2_ photonic crystals by blue luminescent graphene quantum dots (GQDs), n–π* band at ca. 350 nm, is demonstrated as a facile, environmental benign method to promote photocatalytic activity by the combination of slow photon-assisted light trapping with GQD-TiO_2_ interfacial electron transfer. TiO_2_ inverse opal films fabricated by the co-assembly of polymer colloidal spheres with a hydrolyzed titania precursor were post-modified by impregnation in aqueous GQDs suspension without any structural distortion. Photonic band gap engineering by varying the inverse opal macropore size resulted in selective performance enhancement for both salicylic acid photocatalytic degradation and photocurrent generation under UV–VIS and visible light, when red-edge slow photons overlapped with the composite’s absorption edge, whereas stop band reflection was attenuated by the strong UVA absorbance of the GQD-TiO_2_ photonic films. Photoelectrochemical and photoluminescence measurements indicated that the observed improvement, which surpassed similarly modified benchmark mesoporous P25 TiO_2_ films, was further assisted by GQDs electron acceptor action and visible light activation to a lesser extent, leading to highly efficient photocatalytic films.

## 1. Introduction

Structural engineering of semiconductor photocatalysts in the form of photonic crystals (PCs) has been attracting particular attention as an advanced approach to improve solar light harvesting, especially at the absorption edge of wide band gap metal oxides such as titania [1,2]. The underlying mechanism is based on resonant light trapping by periodic macroporous structures, the most common being inverse opals, utilizing the slow photon effect, i.e., light propagation at reduced group velocity for wavelengths near the photonic band gap (PBG) edges [3,4]. Tuning the PBG or stop band (in the case of incomplete PBG) position, which is determined by the inverse opal periodicity, enables overlap of the red- (long-wavelength) or blue-edge (short-wavelength) slow photons of the fundamental bandgap or preferentially higher order slow-light modes [5] with catalyst’s target spectral regions of weak electronic absorbance. This resonance effect can selectively enhance light absorbance at these wavelengths and effectively extend the path length for incident photons promoting photocarrier generation, provided that detrimental stop band (Bragg) reflection losses are moderated. Introduction of TiO_2_ inverse opal photocatalysts in the seminal work of Chen et al. [3] resulted in a marked amplification (enhancement factor of ca. 2.3) of the photocatalytic degradation rate for the methylene blue azo-dye adsorbed on anatase inverse opals with resonant slow photons under both monochromatic UVA and white light irradiation. In addition, the macroporous structure of inverse opals along with the secondary mesoporosity of the nanocrystalline inorganic skeleton offers a porous network of interconnected macro-mesopores [6], which facilitate molecular diffusion and increase the amount of adsorption and reaction sites that are key aspects for the photocatalytic process.

Significant efforts have been accordingly devoted to combine these distinctive structural features with competent compositional modifications of the materials properties such as heterostructuring with noble metal nanoparticles [7,8,9,10], metal oxide nanoclusters [11,12,13], and graphene oxide nanosheets [14,15,16] in order to amplify photocatalytic performance for environmental remediation and solar energy conversion applications. Among the diverse TiO_2_ heterojunction photocatalysts, interfacial coupling of titania with graphene and its derivatives has been an effective method to enhance charge separation and visible light harvesting based on graphene materials’ electron acceptor action and charge transport along with its broadband light absorption [17,18,19]. Graphene quantum dots has been the latest addition to the class of graphene nanomaterials [20], consisting of a graphitic core with sub-10 nm lateral dimensions, whose edges are decorated by oxygenated surface groups, the most abundant being carbonyl, carboxyl, and hydroxyl that improve GQDs dispersion and functionality [21,22]. The exceptional properties of GQDs compared to either plain carbon dots or conventional inorganic QDs, including their size-dependent bandgap and tunable photoluminescence, dictated by quantum confinement and surface/edge functionalization, together with their low toxicity and biocompatibility, have spurred significant interest for a broad range of applications in energy conversion and storage, catalysis, and biomedicine [23,24]. Heterostructured GQD-TiO_2_ photocatalysts, synthesized mainly by the hydrothermal method, have been successfully applied in organic dye degradation under visible light [25,26,27,28,29,30,31,32], especially cationic dyes that readily adsorb on the oxygenated GQDs surface, photoelectrochemical (PEC) solar conversion [33,34], and H_2_ evolution [35,36]. In most cases, the role of GQDs as visible light sensitizer (electron donor) [33,34,37] and/or electron scavenger (acceptor) [35,36] of titania has been related to the enhanced reactivity under visible and UV–VIS light, respectively. The interplay of the two opposite electron transfer pathways depends on GQDs’ size, defect structure, and chemical doping that determine their absorbance in the visible range [26,27,38] as well as the interfacial GQD-TiO_2_ coupling [30], while the controversial upconversion fluorescence of GQDs [39] has been occasionally implicated to account for the visible light activated (VLA) performance [25,28,29]. These variations underline the inherent heterogeneity of GQDs synthesized by different methods and the need for reproducible large–scale production with well-defined characteristics, such as coal’s oxidative cleavage [40], which are major challenges for the use of GQDs in practical applications [24]. Recently, GQDs’ electrodeposition on BiVO_4_ inverse opals, as alternative sensitizers to metallic nanoparticles for PEC water splitting, resulted in a marked rise of the H_2_ evolution rate and photoconversion efficiency [41], while 2.5 nm-sized GQDs deposited on Bi_2_WO_6_ in-situ synthesized on fluorine-doped tin oxide (FTO) inverse opals increased substantially the photocurrent density [42].

In this work, surface functionalization of co-assembled TiO_2_ inverse opals by blue luminescent GQDs is explored as a facile modification method to improve titania’s photocatalytic activity by the synergy of slow photon amplification with GQD-TiO_2_ interfacial electron transfer. To evaluate the materials photocatalytic performance on organic pollutant degradation, salicylic acid (SA) was chosen as a widespread water contaminant that is systematically detected in the influents and effluents of wastewater treatment plants and nearby surface waters due to its extensive application in pharmaceuticals (analgesics) and personal care products [43]. In addition, SA was selected as colorless model pollutant in contrast to organic dyes commonly used for the evaluation of both GQD-TiO_2_ [38] and PC [2] photocatalysts, which are not well suited for the validation of VLA photocatalytic activity due to the self-sensitization effect and the concomitant slow photon effects due to the dyes’ absorbance in the visible range [44]. PBG engineering of the TiO_2_ PCs by tuning the inverse opal void diameter resulted in the distinct rise of the films performance on both SA degradation as well as the increase of photocurrent density under visible and UV–VIS light. This effect was associated with the red-edge slow photons resonating with the composite’s absorption edge, while the concomitant Bragg reflection was attenuated by the GQD-TiO_2_ electronic absorbance in the UVA spectral range. Photoluminescence and PEC results indicated that the enhanced photocatalytic performance, which exceeded that of benchmark mesoporous GQD-P25 titania films, was also related to the improved charge separation at the GQD-TiO_2_ interface as well as to the action of GQDs as VLA sensitizers of titania nanocrystals.

## 2. Materials and Methods

### 2.1. Films Deposition and GQDs Surface Modification

Monodisperse polystyrene (PS) spheres with mean diameters of 211, 300, and 340 nm as well as poly(methyl methacrylate)-PMMA ones of 261 nm diameter were purchased from Microparticles GmbH (Berlin, Germany) in the form of colloidal dispersion of 5% solids (w/v) in deionized water (6–10 nm standard deviation and 2.4–3.5% CV). Titanium(IV) bis(ammonium lactato)dihydroxide (TiBALDH) 50 wt.% aqueous solution (388165, Sigma-Aldrich, St. Louis, MO, USA) and coal-derived QGDs (900708, Sigma-Aldrich, St. Louis, MO, USA) in the form of 1 mg/mL suspension in H_2_O with nominal diameter < 5 nm and topographic height of 1–2.0 nm were obtained from Sigma Aldrich. All other reagents were of analytical or ACS reagent grade.

Titania inverse opals were deposited on glass slides via the evaporative co-assembly of polymer colloidal spheres with the hydrolyzed TiBALDH titania precursor [45]. In this case, liquid phase infiltration by water-soluble sol–gel precursors is selected as a low-cost and scalable fabrication alternative to conformal, though more expensive and time-consuming vapor phase deposition methods (e.g., atomic layer deposition and chemical vapor deposition), while the conventional successive colloidal template self-assembly and infiltration processes are combined into a single-step that may lead to more robust PC films [9,45]. The glass substrates after cleaning with Hellmanex™ III (Z805939, Sigma-Aldrich, St. Louis, MO, USA), ultrasound, and acetone (32201, Honeywell Riedel-de Haën, ACS reagent, ≥99.5%, Seelze, Germany)—EtOH (32221, Honeywell Riedel-de Haën, puriss. p.a., absolute, ≥99.8%, Seelze, Germany) washing, were almost vertically suspended into beakers containing 10 mL of a 0.125 wt.% polymer sphere suspension and 0.07 mL of fresh titania precursor prepared by 0.25 mL of TiBALDH, 1 mL of EtOH and 0.5 mL of 0.1 M HCl (30721 Fluka, ACS reagent, ≥37%). After solvent evaporation at 55 °C, the dry films comprising the infiltrated titania gel within the opal interstices were calcined at 500 °C for 1 h in air (1 °C/min), to remove the polymer matrix and crystallize TiO_2_ in the inverse opal structure. The plain TiO_2_ PC films were designated as PCXXX, with XXX = 211, 261, 300, and 340 being the templating sphere diameter. Surface modification by GQDs was performed by immersion of the TiO_2_ films in the aqueous GQDs suspension (sonicated for 15 min prior to use) under dark conditions for 24 h. The GQDs-impregnated TiO_2_ films were subsequently washed in deionized water and dried under N_2_ flow with no further treatment. It should be noted that impregnation for longer times resulted in GQD aggregation and decrease of the photocatalytic performance and thus was only used for spectroscopic identification of GQDs on the TiO_2_ PCs (vide infra). The surface-modified PC films were named “GQD-PCXXX”.

Mesoporous TiO_2_ reference films were deposited on cleaned glass slides by spin coating at 1000 rpm for 60 s paste of the benchmark Aeroxide^®^ P25 mixed phase titania nanocatalyst [46] in order to validate the photonic films’ performance. After drying at 120 °C (15 min), the films were annealed at 450 °C for 30 min in air and were designated as P25. The deposition was repeated twice in order to increase the film thickness. The mesoporous films were subjected to GQDs’ surface modification under identical conditions with the photonic films and were accordingly named “GQDs-P25”.

### 2.2. Materials Characterization

The films morphology and phase composition were investigated by scanning electron microscopy (SEM, Quanta Inspect, FEI, Eindhoven, Netherlands) along with energy-dispersive X-ray analyzer (EDXDX4, EDAX, Mahwah, NJ, USA) and a FEI Talos F200i field-emission (scanning) transmission electron microscope (Thermo Fisher Scientific Inc., Waltham, MA, USA) operating at 200 kV, equipped with a windowless energy-dispersive spectroscopy microanalyzer (6T/100 Bruker, Hamburg, Germany). The films were characterized by micro-Raman spectroscopy (inVia Reflex, Renishaw, London, UK) using 514 nm excitation. The laser beam was focused using a 100 × (ΝA = 0.9) objective at low power density (0.1 mW/μm^2^) to avoid local heating. X-ray photoelectron spectroscopy (XPS) was performed on a PHOIBOS 100 (SPECS, Berlin, Germany) hemispherical analyzer using non-monochromatized Mg-Ka radiation (1253.6 eV) of the SPECS XR50 source. The spectrometer was calibrated using clean silver, copper, and gold. The Ag *3d*_5/2_, Cu *2p*_3/2_, and Au *4f*_7/2_ peak positions were determined at 368.3, 932.7, and 84 eV, respectively. The XP spectra were collected at take-off angle of 52° using pass energy of 7 eV. The adventitious C 1s set to 284.8 eV was used for charge referencing [47]. Fitting was made using XPS Peak Fit and Sirley background subtraction.

Photoluminescence (PL) measurements were carried out using the focused beam of a 275 nm light-emitting diode for excitation, and the PL signal was collected though a long-pass 320 nm cutoff filter by a fiber optic spectrophotometer (LR1, ASEQ Instruments, Vancouver, Canada). The optical properties were investigated by specular and diffuse reflectance UV–visible (VIS) spectroscopy (Cary 60, Agilent, Santa Clara, CA, USA) equipped with fiber-optic diffuse reflectance (Barrelino) and 15° specular reflectance (PIKE, UV-Vis 15Spec, PIKE Technologies, Madison, WI, USA) accessories, using a Halon standard and a UV-enhanced Al mirror for baseline measurements.

Photoelectrochemical characterization was carried out in a standard three-electrode system employing an Autolab PGSTAT302N (EcoChemie, Utrecht, The Netherlands) potentiostat, a Pt foil as counter, and Ag/AgCl as reference electrodes. The working electrode was prepared by PC and P25 deposition on cleaned fluorine-doped tin oxide (FTO) conductive glass (FTO 7 ohms/sq, Sigma-Aldrich, St. Louis, MO, USA) followed by GQDs surface modification. The electrolyte was 0.5 M NaOH, while UV–VIS illumination was provided by a 300 W Xe lamp in combination with an AM 1.5 G filter for simulated solar light (100 mW/cm^2^). Electrochemical impedance spectroscopy (EIS) measurements were carried out at open-circuit voltage (V_OC_), in the frequency range of 10^4^–10^−1^ Hz with ac amplitude of 10 mV.

### 2.3. Photocatalytic Performance

The films photocatalytic activity was tested on the aqueous phase degradation of salicylic acid (SA, 247588, Sigma-Aldrich) under UV–Vis and Vis light. Films of 2 cm^2^ were placed horizontally in vials containing aqueous SA (4 mL, 35 μΜ) solution, where they were stirred for 30 min under dark conditions to reach adsorption–desorption equilibrium. To enhance SA adsorption on the TiO_2_ films, the solution pH was stabilized at 3 by dilute HCL. The UV–VIS illumination source was a 150 W Xe lamp (6255, ORIEL GmbH, Darmstadt, Germany) along with a 305 nm cutoff filter (20CGA-305, Newport, RI, USA) and a heat-reflective mirror (20CLVS-3 CoolView™, Irvine, CA, USA). Visible light was selected by 400 nm cutoff filters (20CGA-400, Newport, RI, USA). The horizontal beam was directed on the film surface by UV-enhanced Al mirror (ValuMax 20D520AL.2, Newport, RI, USA) at incident power density of 96 mW/cm^2^. A 0.5 mL aliquot was periodically withdrawn from the SA solution and analyzed in the spectrophotometer. Additional photocatalytic tests were carried out at pH values of 7 and 10 as well as in the presence of 0.01 M of methanol (MeOH, 179337, Sigma-Aldrich, ACS reagent, ≥99.8%) as hydroxyl radical (^•^OH) scavenger [48]. The tests were performed in triplicate, and standard errors were calculated for the mean kinetic constants.

## 3. Results and Discussion

### 3.1. Film Structure, Phase Composition, and Optical Properties

**Figure 1:**
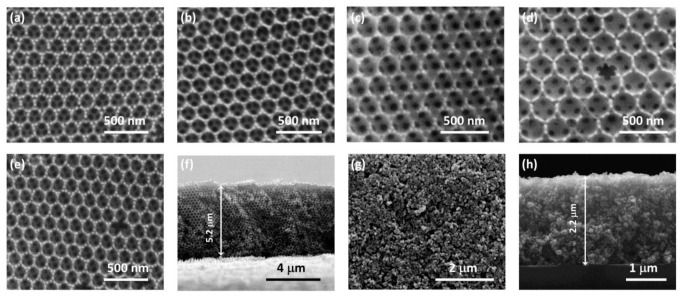
Top-view SEM images of (**a**) PC211, (**b**) PC261, (**c**) PC300, (**d**) PC340, (**e**) GQD-PC211 inverse opals, and (**f**) cross-section image of PC340. Top-view (**g**) and cross-section (**h**) images of P25 films.

Figure 1a–d shows representative top-view SEM images of the PC films, co-assembled from polymer spheres of different diameters. Well-ordered inverse opal structures with thickness of 5.2(5) µm (Figure 1f) were invariably observed, displaying a periodically structured network of void macropores with variable size, interconnected through smaller ones (dark circular areas within the large macropores) that form at the contact points of adjacent polymer spheres after calcination. The mean void diameters (*D*) determined by SEM, increased proportionally to the templating sphere size (Table 1) as a result of the amorphous to crystalline titania phase transition and the associated volume shrinkage after calcination [49]. It should be noted that the degree of contraction was about 37% in the case of PS spheres and about 41% for the PMMA (PC261) ones, which were more resilient to calcination. The P25 films exhibited a rough, sponge-like morphology, typical of mesoporous films (Figure 1g). Their thickness was approximately 2.2 µm (Figure 1h) resulting in appreciably higher, by at least 50%, titania mass loading compared to the macroporous inverse opals assuming identical mesoporosity and an ideal titania filling factor of 0.26 for the PC films (vide infra).

**Figure 2:**
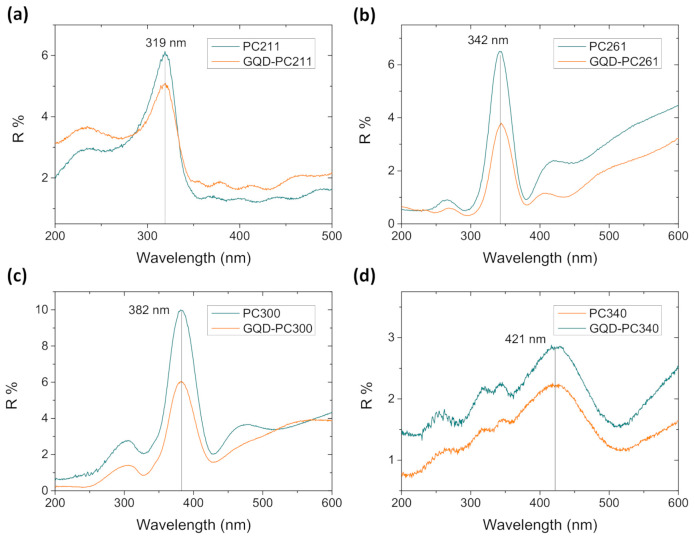
Specular reflectance (R%) spectra for the (**a**) PC211, (**b**) PC261, (**c**) PC300, and (**d**) PC340 photonic films before and after QGDs surface modification at 15° incidence angle.

PBG formation was identified for all PC films by the specular reflectance (R%) spectra at 15° incident angle, as shown in Figure 2. A distinct R% peak due to Bragg reflection was observed at increasing wavelengths with the macropore size (Table 1), characteristic of the stop band formation along the [111] direction in TiO_2_ inverse opals. Fabry-Pérot interference fringes were also detected outside the stop band spectral range, indicating relatively uniform photonic domains within the area of the probe beam (<1 mm^2^).

The stop band positions can be described by modified Bragg’s law for first-order diffraction from the (111) planes of an *fcc* lattice of spherical void macropores [2]:

$$\lambda =2d_{111}\sqrt{n_{eff}^{2}-\sin^{2}\theta },$$

where *λ* is the stop band wavelength, $d_{111}=\sqrt{2/3}D$ is the spacing between (111) planes, and $n_{eff}^{2}=n_{\mathrm{void}}^{2}f+n_{{\mathrm{TiO}}_{2}}^{2}\left(1-f\right)$ is the volume-weighted average of the void spheres’ refractive index ($n_{\mathrm{void}}$) and titania ($n_{{\mathrm{TiO}}_{2}}$) that occupies the inverse opal skeleton, while *f* is the void filling fraction (*f* = 0.74 for the ideal *fcc* lattice) and *θ* is the angle between the incident beam and the plane normal.

Applying modified Bragg’s law for the experimental stop band wavelengths λ_exp_ (15°) at *θ* = 15° together with the measured diameters *D* for $n_{{\mathrm{TiO}}_{2}}=2.55$ and $n_{\mathrm{air}}=1.0$, the $n_{eff}$ values and solid filling fractions (1 − *f*) were determined in air (Table 1). Moreover, using the obtained filling fractions and $n_{H_{2}O}=1.33$, the stop band positions were estimated in water (Table 1), where the photocatalytic reaction takes place. The derived 1−f were smaller than the theoretical value of 0.26 for complete filling of the inverse *fcc* lattice and increased systematically with the decrease of macropore size. It should be noted that although the obtained 1−f values provide only a rough estimate based on modified Bragg’s law, they agree with those obtained by rigorous theoretical simulations of R% spectra for TiO_2_ PCs [16]. Moreover, the observed increase of the filling fraction for the smaller macropores complies favorably with recent results on co-assembled TiO_2_ PC films [13]. In that case, an analogous increase of surface area and mesopore volume was detected, related to the enhanced mesoporosity of the inverse opal skeletal walls due to the increase of available interfaces for the smaller macropores [50].

**Figure 3:**
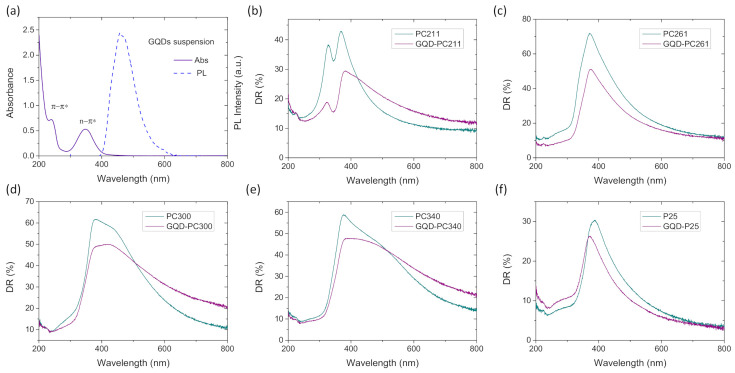
(**a**) Absorbance and photoluminescence (PL) of the graphene quantum dot (GQD) suspension (diluted to 0.025 mg/mL). Diffuse (DR%) reflectance spectra for (**b**) PC211, (**c**) PC261, (**d**) PC300, (**e**) PC340, and (**f**) P25 films before and after QGDs surface modification.

Surface modification by GQDs, which had no effect on the inverse opal macropore structure (Figure 1e), resulted in the systematic moderation of the R% peak intensity for all PC films (Figure 2) reflecting the contribution of GQDs’ electronic absorbance, especially in the UVA range. Figure 3a displays the UV–VIS absorption spectrum of dilute (0.025 mg/mL) aqueous GQD suspension, where two bands at 240 and 347 nm were clearly detected, associated with the π–π* and n–π* transitions of C=C and C=O bonds in the GQDs, respectively, along with the characteristic blue PL emission at 456 nm [24,39,40]. The GQDs influence was also identified in the diffuse reflectance (DR%) spectra of the QGD surface-modified PC and P25 films, as shown in Figure 3b–f. In that case, an intense, broad DR% band was observed for the pristine PC211, PC300, and PC340 films and a weak shoulder for PC261, following closely the diameter-dependent PBG position (Table 1), in contrast to the constant anatase absorption edge at about 380 nm and the featureless spectra of the mesoporous mixed-phase P25 titania films at *λ* > 400 nm. The width and intensity of the DR% Bragg peak were considerably higher than the corresponding R% ones indicative of increased scattering due to film areas with uneven surfaces of different flatness and thickness within the spot of the coarse beam used for the DR% measurement. In addition, the DR% Bragg peak was systematically shifted to higher wavelengths compared to the corresponding R% ones. This effect cannot be explained only by the 15° incidence but also because red slow photons, which are localized at the inorganic skeleton, experience a longer optical path and thus lead to increased scattering [15]. The DR% intensity was appreciably reduced after GQDs’ deposition due to the extended GQDs absorbance spectrum (Figure 3a), especially below 400 nm, similar to the effect of graphene oxide nanocolloids decorating TiO_2_ PCs [15]. This was most evident for the PC211 films, where the stop band DR% peak was clearly detected at the side of the n–π* GQDs absorbance band, corroborating the presence of QGDs on the titania films.

**Figure 4:**
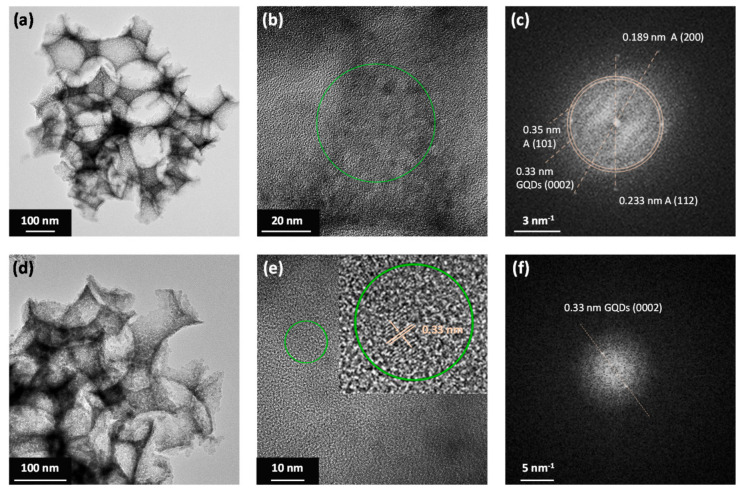
(**a**,**b**,**d**,**e**) TEM images of the QGD-PC261 film at different magnifications. (**c**,**f**) show the fast Fourier-transform (FFT) patterns of the areas indicated by circles in the high-resolution TEM images of (**b**,**e**), respectively.

The phase composition of the PC films was investigated by TEM analysis, as shown in Figure 4.

TEM images of the GQD-PC261 films at different magnifications confirmed that the inverse opal skeleton was mesoporous consisting of aggregated, nanocrystalline particles (Figure 4a,d) with distinct *d*-spacings, the most common being the 0.35 nm one, corresponding to the (101) planes of the anatase TiO_2_ phase, as evidenced by the fast Fourier-transform (FFT) patterns of the broad circled area of Figure 4b. Τhe presence of the anatase phase could be readily verified at additional spots corresponding to the *d*-spacings of the (200) and (112) anatase planes, while GQDs could be also identified from the (0002) graphitic planes with 0.33 nm spacing [51], which is very close to the (101) anatase ones (Figure 4c). Moreover, it was possible to trace isolated diffraction spots on the titania walls corresponding to 0.33 nm and thus confirm QGDs deposition, as shown in Figure 4e,f.

**Figure 5:**
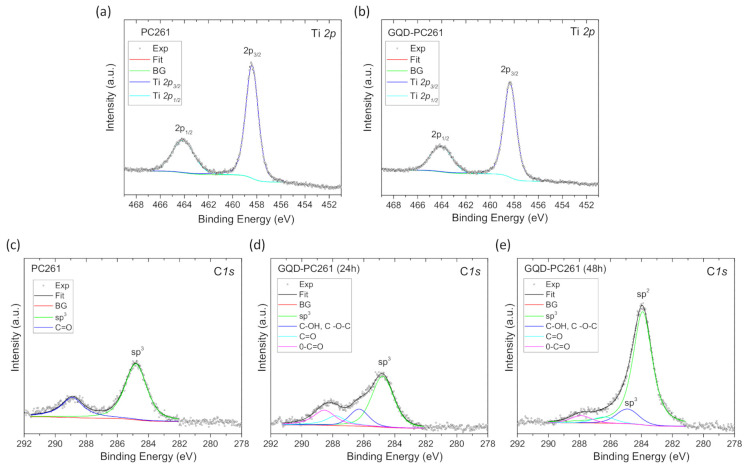
Ti 2p X-ray photoelectron XP spectra for the (**a**) PC261 and (**b**) GQD-PC261 films. C 1s XP spectra for the (**c**) PC261 and (**d**,**e**) GQD-PC261 films after surface modification for 24 h (48 h) impregnation. Solid lines show the total fit and the individual peak deconvolution. The *sp^3^* component in (**c**,**d**) is attributed to adventitious carbon, while in (**e**) the *sp^2^* component indicates GQDs presence.

The surface composition of the GQD–PC films was also explored by XPS. Figure 5a,b shows the characteristic Ti *2p* spin-orbit doublets for the pristine and GQD-modified PC261 inverse opals. Fitting of the experimental Ti 2p_3/2_ and Ti 2p_1/2_ peaks shows a contribution at ~458.4 eV with full-width at half-maximum (FWHM) of ~1.2 and 464.12 eV with FWHM~2.1 eV, respectively, with a separation of 5.72 eV. These values indicate the presence of Ti^4+^ ions and stoichiometric TiO_2_ [52] in good agreement with literature [45]. The FWHM values are slightly wider those in Biesinger et al. [45] due to the non-monochromatized source. Typically, the FWHM for each spin-orbit component is the same, but for Ti 2p, the Ti 2p_1/2_ is much broader than the Ti 2p_3/2_ peak, indicating that no other Ti contribution can be resolved. Figure 5c,d shows the characteristic C 1s peak and the respective peak deconvolution for the PC261 and GQD-PC261 films, where a dominant peak at 284.8 eV and a weaker one at 288.8 eV were observed for the unmodified film, indicative of adventitious *sp^3^* carbon deposits and carbonyl (C=O) surface groups, respectively, in the macroporous film [25,53]. These signals varied weakly after GQDs deposition; the most prominent changes were a shift of the C=O peak at ~288.1 eV and an increase of spectral weight above 286 eV, which might be related to epoxy/hydroxyl groups (C–O) [47]. To clearly attest GQD’s presence on the titania inverse opals, XP C1s spectra were recorded on GQD-PC261 films modified by 48 h impregnation in order to increase GQDs loading (Figure 5e). In that case, the C1s peak was markedly intensified and shifted to lower binding energy of 284.0 eV confirming the presence of *sp^2^* species.

**Figure 6:**
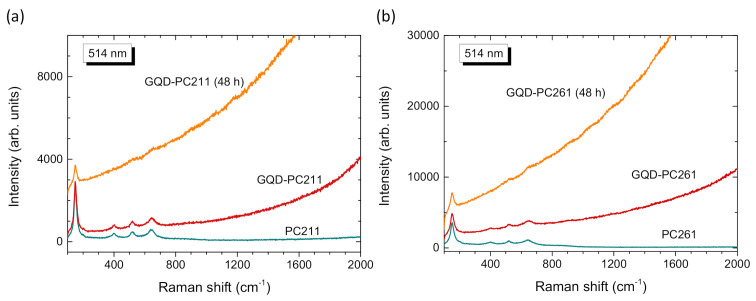
Raman spectra of the (**a**) PC211 and (**b**) PC261 films before and after GQDs’ surface modification at 514 nm.

The phase composition of the GQD-TiO_2_ PCs was further investigated by Raman spectroscopy. Figure 6 shows representative Raman spectra for the PC films before and after QGDs modification at 514 nm. The inverse opals exhibited the characteristic Raman-active modes of the anatase titania phase at approximately 148 (E_g_), 199 (E_g_), 399 (B_1g_), 519 (A_1g_ + B_1g_), and 642 cm^−1^ (E_g_). No traces of polymeric species or other TiO_2_ phases were detected, confirming that the inverse opals crystallized in the single anatase phase after calcination at 500 °C, in agreement with the TEM analysis. Moreover, appreciable broadening and shift of the anatase Raman modes was detected, especially for the most intense low-frequency E_g_ mode, which shifted to 148 cm^−1^ and broadened to a full-width at half-maximum (FWHM) of 19 cm^−1^, indicative of the breakdown of the *q* = 0 selection rule for Raman scattering [54]. The formation of ca. 8 nm anatase nanoparticles can be accordingly predicted from the frequency vs. width correlation curves of the E_g_ mode [54], in agreement with recent results on the formation of sub-10 nm anatase nanocrystals in co-assembled TiO_2_ inverse opals using the TiBALDH precursor [13,15]. Furthermore, no Raman mode such as the G band arising from the stretching of sp^2^ carbon atoms and the highly dispersive D-band activated by defects [15,16,40], could be traced for the GQD-PC films in the frequency range of 1000–2000 cm^−1^. However, GQD’s deposition on the titania PC films resulted systematically in the upsurge of an intense PL background, most pronounced for the GQD-PC films after 48 h impregnation, correlating with the variation of the GQDs loading amount. This feature can be well accounted by the intense PL emission of GQDs [24], which corroborates their presence on the anatase PC skeleton, though it masks their Raman modes.

### 3.2. Photocatalytic-PEC Performance and Charge Separation

**Figure 7:**
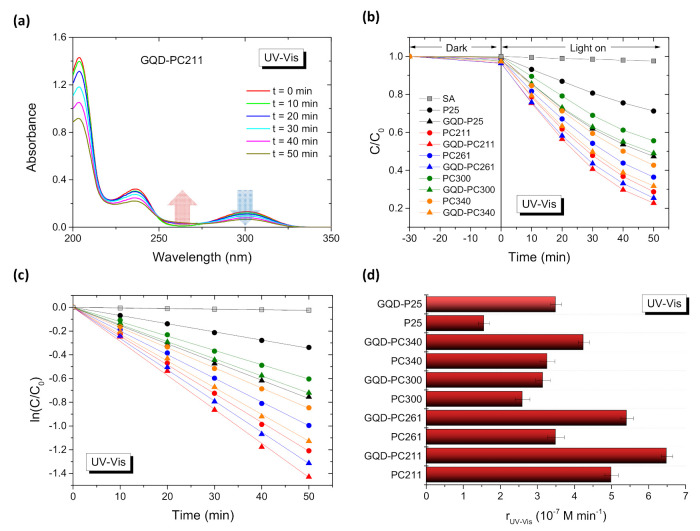
Salicylic acid (SA) (**a**) absorbance spectra, (**b**,**c**) photodegradation kinetics, (**d**) and reaction rates for the pristine and GQDs surface-modified PC and P25 TiO_2_ films under UV–VIS light irradiation. Solid lines in (**c**) correspond to the best fit curves of the ln(*C*/*C*_0_) vs. *t* plots.

**Figure 8:**
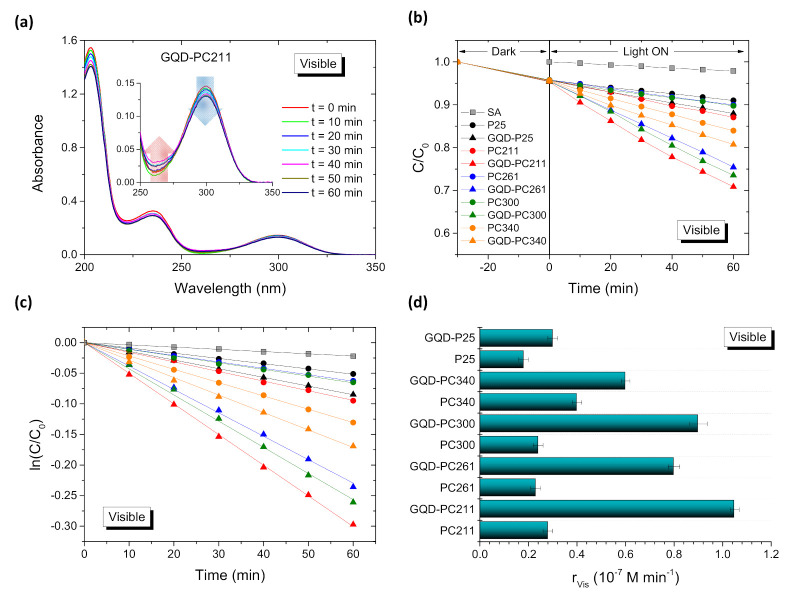
Salicylic acid (SA) (**a**) absorbance spectra, (**b**,**c**) photodegradation kinetics, and (**d**) reaction rates for the pristine and GQDs surface-modified PC and P25 TiO_2_ films under visible light irradiation. Solid lines in (**c**) correspond to the best fit curves of the ln(*C*/*C*_0_) vs. *t* plots.

The photocatalytic activity of the photonic and P25 reference GQD-TiO_2_ films was evaluated on the degradation of salicylic acid (SA) as model water pollutant under UV–VIS and visible light, as shown in Figure 7 and Figure 8, respectively. SA is a colorless water contaminant, which, unlike dye pollutants, absorbs well below the PC’s stop bands in the UV range and thus prevents any slow photon contribution via spectral overlap with the molecular electronic absorption [15,47]. The photodegradation tests were carried out at acidic pH = 3 that assists SA chemisorption on titania and drives direct SA oxidation by valence band holes [55]. Blank experiments in the absence of photocatalysts, indicated negligible SA degradation under UV–VIS and visible light, whereas illumination in the presence of the pristine and GQD-TiO_2_ films, after dark adsorption, resulted in the continuous temporal decrease of the SA concentration (*C*) (Figure 7a,b and Figure 8a,b), which was determined spectrophotometrically by the characteristic SA absorption band at 300 nm [56]. Besides the continuous decrease of the main SA bands with time, a weak absorbance increase was traced at about 260 nm (depicted by arrows in Figure 7a and Figure 8a). This behavior indicates the formation of intermediate products during the photocatalytic reaction, which may be attributed mainly to dihydroxybenzoic acids and linear short-chain carboxylic acids [57,58].

In all cases, the ln(*C*/*C*_0_) vs. *t* plots varied linearly (Figure 7c and Figure 8c) indicating that SA photodegradation followed pseudo first-order kinetics under both illumination conditions. The apparent kinetic constants $k_{\mathrm{UV}-\mathrm{Vis}}$ and $k_{\mathrm{Vis}}$ were accordingly derived from the slopes of the linear regression curves of the ln(*C*/*C*_0_) vs. *t* plots. To determine the films’ photocatalytic activity independently of variations of the concentration $C_{0}$ after dark adsorption, the reaction rates, $r_{\mathrm{UV}-\mathrm{Vis}}$ and $r_{\mathrm{Vis}}$, were calculated as $r=kC_{0}$, which applies well for low (<mM) pollutant concentrations. The obtained reaction rates varied significantly depending on the macropore diameter of the inverse opals and the illumination conditions, as shown in Figure 7d and Figure 8d, indicative of considerable differences in the light-harvesting ability of the PC films.

More importantly, GQDs deposition resulted invariably in the improvement of both reaction rates $r_{\mathrm{Vis}}$ and $r_{\mathrm{UV}-\mathrm{Vis}}$, most pronounced under visible light (*λ* > 400 nm), where the films VLA performance is compromised by the inherently weak electronic absorbance of both anatase PC and mixed-phase P25 films [13]. Under UV–VIS illumination, a clear maximum of $r_{\mathrm{UV}-\mathrm{Vis}}$ was observed for both pristine and QGD-PC211 films (Figure 7d), followed by the corresponding pristine and modified PC261. This diameter-dependent variation can be related to the slow photon amplification for PC211, whose stop band is expected at about 367 nm in water (Table 1). According to the FWHM of the Bragg R% peak (Figure 2) and rigorous theoretical simulations of the R% spectra for anatase PC films [16], the stop band’s spectral width without the contribution of unresolved Fabry-Pérot fringes, can be estimated to be of ca. 40 nm. This leads to a spectral range of about 390–410 nm for the PC211 red-edge slow photons, which matches closely the anatase electronic absorption edge and the tail of GQDs n–π* band (Figure 3a), while the Bragg reflectance is attenuated by the strong UVA absorbance of both anatase nanocrystals and GQDs. In this case, the GQDs optical response in the UVA range follows closely anatase’s absorbance and thus acts synergistically to the slow photon enhancement mechanism for the GQD-TiO_2_ PC films. The $r_{\mathrm{UV}-\mathrm{Vis}}$ rates decreased for the PC261 films, whose stop band is expected at ca. 400 nm in water (Table 1), while a relative increase of $r_{\mathrm{UV}-\mathrm{Vis}}$ was noted for the PC340 films, which might be related to their larger macropores [59,60]. Comparing the $r_{\mathrm{UV}-\mathrm{Vis}}$ values for the best performing PC211 films with PC300 and PC340, enhancement factors of about 1.9 and 1.5 for the efficiency of the photonic amplification were obtained. An additional increase by 30–55% is observed for the $r_{\mathrm{UV}-\mathrm{Vis}}$ rates of the QGD-PC films compared to the pristine ones after GQDs functionalization. Slow photon effects at the GQD-TiO_2_ absorption edge and the concomitant diameter dependence of $r_{\mathrm{UV}-\mathrm{Vis}}$ were appreciably moderated under visible light (Figure 8d). In that case, GQDs’ deposition resulted in the marked enhancement of the VLA performance, i.e., $r_{\mathrm{Vis}}$ increased by 3–4 times compared to the unmodified PC films, corroborating GQDs action as broadband visible light sensitizer of titania. However, the obtained $r_{\mathrm{Vis}}$ rates were much smaller than the $r_{\mathrm{UV}-\mathrm{Vis}}$ ones, indicating significantly lower process efficiency compared to UV–VIS activation.

**Figure 9:**
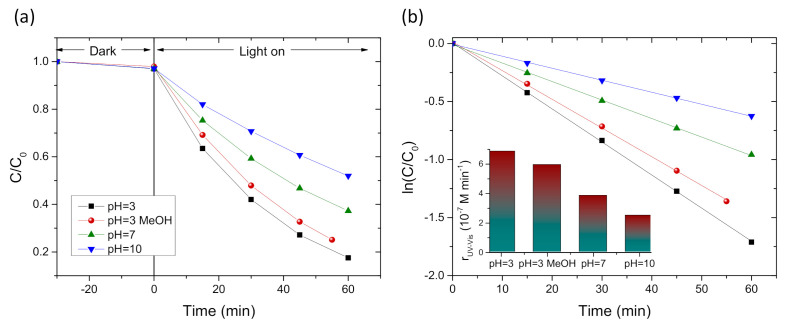
SA photodegradation kinetics by GQD-PC211 films under UV–VIS light for pH values of 3, 7, and 10 and in the presence of 0.01 M MeOH at pH = 3. (**a**) ln(*C*/*C*_0_) vs. *t*; (**b**) ln(*C*/*C*_0_) vs. *t* plots. The inset in (**b**) shows the corresponding reaction rates.

To further investigate the SA degradation mechanism, comparative photocatalytic tests were performed at pH values of 7 and 10 for the QGD-PC211 films under UV–VIS light, as shown in Figure 9a. The increase of pH resulted in the continuous decrease of $r_{\mathrm{UV}-\mathrm{Vis}}$ (inset of Figure 9b), in agreement with previous reports [55,57]. This variation supports the major role of acidic pH in the formation of SA-TiO_2_ surface complexes via bidentate binding of salicylate to single undercoordinated Ti(IV) ions on titania [16,61] and subsequent oxidation by valence band holes [62]. The latter effect was further explored by photodegradation tests in the presence of MeOH as ^•^OH scavenger under identical conditions at Ph = 3. In that case, MeOH had little influence on the SA degradation kinetics, indicating that photogenerated holes are the main reactive species in SA oxidation at low pH.

**Figure 10:**
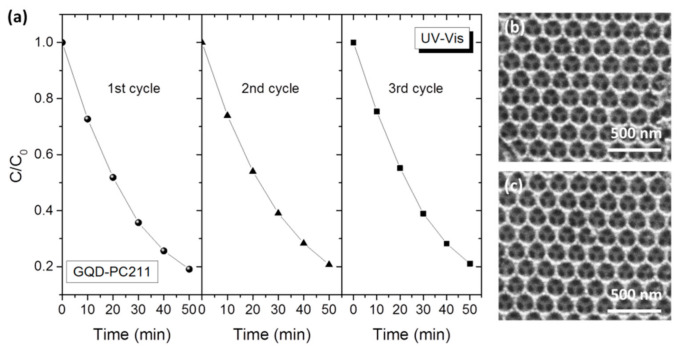
(**a**) SA photodegradation kinetics for three successive tests using the same GQD-PC211 film under UV–VIS light; SEM images of GQD-PC261 (**b**) before and (**c**) after SA photodegradation.

The stability of the best performing QGD-PC211 inverse opal was evaluated by three successive SA photocatalytic cycles using the same film under UV–VIS light with intermediate cleaning of SA residues under additional 1 h UV–VIS illumination in 3 mL of deionized water. The QGD-PC211 films showed excellent stability (the reaction rates varied within 6–8%) after three cycles, as shown in Figure 10a. Moreover, SEM images of QGD-PCs after SA photocatalytic test (Figure 10b) indicated that the films retained intact the inverse opal structure, corroborating the chemical stability of GQDs-TiO_2_ after aqueous-phase organic pollutant photodegradation.

**Figure 11:**
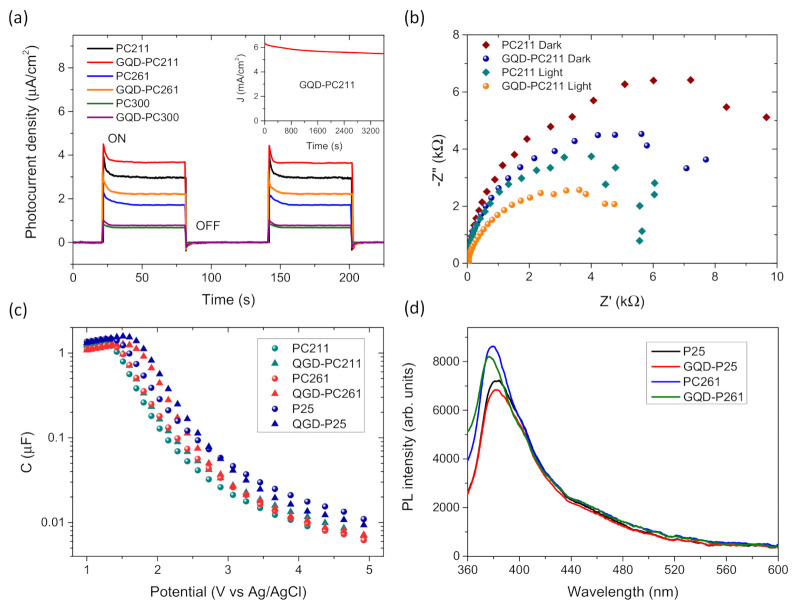
(**a**) Transient photocurrent density for pristine and surface-modified GQD-PC films under UV–VIS illumination; (**b**) EIS Nyquist plots for the PC211 and GQD-PC211 films under dark and UV–VIS illumination; (**c**) capacitance vs. applied potential for pristine and GQDs-modified PC and P25 films at 1000 Hz; (**d**) PL spectra for P25 and PC211 before and after GQDs surface modification. The inset in (**a**) shows the variation of photocurrent density (J) for GQD-PC211 films under continuous UV–VIS irradiation for 1 h at 1.23 V Ag/AgCl.

Photocurrent generation was subsequently investigated for the GQD-PC films deposited on FTO glass substrates under chopped UV–VIS illumination from a 300 W Xe lamp, as shown in Figure 11a. The photocurrent transients exhibited the characteristic spikes stemming from the flux of photogenerated holes to the semiconductor–electrolyte interface, followed by an exponential decay to a steady-state value, reached through surface recombination with conduction band electrons [63]. The PC211 films presented the highest photocurrent density complying favorably with the enhanced slow photon light trapping for this macropore diameter. Moreover, GQDs surface modification resulted in further amplification of the photocurrent density for all GQD-PC films, about 25% for the QGD-PC211 ones, following closely the enhancement of the UV–VIS photocatalytic performance (Figure 7). Moreover, the latter films presented high photocurrent stability during continuous UV–VIS irradiation for 1 h at 1.23 V Ag/AgCl, as shown in the inset of Figure 11a.

To explore GQD-TiO_2_ interfacial charge transfer, EIS measurements were performed on the PC211 films exhibiting the highest photocatalytic activity, before and after GQDs surface modification, at V_OC_ under both dark and UV–VIS light. Figure 11b shows the Nyquist plots corresponding to the imaginary part *Z″* vs. the real part *Z′* of the complex impedance *Z*, where a well-defined capacitive arc of variable curvature was obtained at higher frequencies in the EIS plane. GQDs deposition resulted in the decrease of impedance and the corresponding arc-radius under both dark and light illumination conditions, indicating lower charge transfer resistance and thus reduced recombination kinetics for the GQD-TiO_2_ films. Assuming a single-arc behavior, the system’s capacitance *C* was also derived from the imaginary part of *Z* at 1000 Hz as a function of the applied voltage under UV–VIS illumination, as shown in Figure 11c. Increased capacitance was observed for both GQD-PC and GQD-P25 films at lower potentials, indicating higher charge accumulation at the GQD-TiO_2_ interface under UV–VIS light, in line with thorough electrochemical studies of surface-modified TiO_2_ by GQDs [34] and reduced graphene oxide sheets [64].

Charge separation was further investigated by PL spectroscopy for the GQD-PC261 and GQD-P25 films under 275 nm excitation, as shown in Figure 11d. The PL spectra of the unmodified PC and P25 films showed a broad band at about 380 nm arising from the near-band gap PL emission of the titania nanocrystals with small variations between the single-anatase PC and anatase/rutile P25 films, accompanied by much weaker shoulders at *λ* > 400 nm originating from shallow defect states [65,66], though with no sign of GQD’s blue emission. Surface modification of the PC and P25 films by GQDs resulted in the decrease of PL intensity corroborating the reduced electron–hole recombination and most importantly, pointing to the interfacial transfer of UV photogenerated electrons from TiO_2_ to GQDs that function as electron scavengers. It can be accordingly concluded that the enhanced photocatalytic performance of the GQD-PC films under UV irradiation is primarily due to the improved charge separation arising from electron transfer from TiO_2_ to the GQDs, whereas a minor contribution accounting for the weaker VLA photocatalytic performance arises from GQDs action as electron donor (sensitizer) of TiO_2_ under visible light [35,67]. Combination of GQD-TiO_2_ interfacial electron transfer with the slow photon-assisted light harvesting of TiO_2_ inverse opals can be a promising route to enhance light harvesting and lessen electron–hole recombination for the development of efficient photocatalytic films.

## 4. Conclusions

Surface modification of TiO_2_ photonic crystals in the form of anatase inverse opals has been implemented by impregnation in aqueous suspension of coal-derived, blue luminescent GQDs with n–π * absorption band at about 350 nm. PBG engineering of the co-assembled inverse opal films to the GQDs-TiO_2_ composite’s absorption edge resulted in distinct improvements of the SA photocatalytic degradation and photocurrent density under UV–VIS and visible light illumination, outperforming benchmark mesoporous GQD-P25 films subjected to the same treatment. The observed amplification was related to the enhanced light harvesting by red-edge slow photons resonating with GQD-TiO_2_ absorption edge, while Bragg reflection losses were mitigated by the composite’s UVA electronic absorbance. Synergistic slow photon enhancement could be thus attained as GQDs’ UVA absorbance matched closely the electronic band gap of the anatase nanocrystals forming the inverse opal skeleton. Photoelectrochemical and photoluminescence measurements indicated that the photocatalytic performance was mainly assisted by GQDs’ action as electron acceptors, while a smaller contribution was inferred by the action of GQDs as visible light sensitizer. The obtained results support that judicious combination of the slow photon-assisted light harvesting of TiO_2_ PCs with the tunable GQDs electronic absorbance and GQD-TiO_2_ interfacial electron transfer can be a promising, environmentally benign approach to improve charge separation and light harvesting of titania photocatalytic films. The benefits of PBG-engineered GQD-TiO_2_ PC photocatalysts can be further leveraged to other photo-induced applications besides water pollutant degradation, especially those based on the stabilization of efficient meso-macroporous photocatalytic materials. In particular, they could be fruitfully applied in air purification from volatile organic compounds and NO_x_, PEC water splitting, and H_2_ production as well as in the development of nanostructured semiconductor substrates for surface-enhanced Raman scattering (SERS) based on the unique combination of charge transfer and electromagnetic enhancement via slow photon tuning that may serve as cost-effective and recyclable alternatives to conventional coinage metal SERS substrates.

## Figures and Tables

**Table 1:** Structural and optical parameters of the TiO_2_ photonic crystal (PC) films.

Film	*D*^1^ (nm)	Variation % ^2^	λ_exp_ (15°) ^3^ (nm)	*n*_eff_ (air)	1 − *f*	*n*_eff_ (H_2_O)	λ(0°) ^4^ (H_2_O)
PC211	135(7)	64(4)	319	1.47(7)	0.21(4)	1.66(8)	367
PC261	153(5)	59(2)	342	1.39(4)	0.17(2)	1.61(5)	401
PC300	190(5)	63(3)	382	1.26(3)	0.11(1)	1.51(3)	467
PC340	215(5)	63(2)	421	1.23(3)	0.09(1)	1.48(3)	521

^1^*D*: macropore diameter determined by SEM. ^2^ Macropore variation: D/D_sphere_^3^ λ_exp_(15°): stop band position determined from the 15° incidence R% spectra. ^4^ λ(0°): PBG position calculated from modified Bragg law at *θ* = 0°.
